# Priority setting in a context of insecurity, epidemiological transition and low financial risk protection, Afghanistan

**DOI:** 10.2471/BLT.18.218941

**Published:** 2019-04-01

**Authors:** Karl Blanchet, Feroz Ferozuddin, Ahmad Jan J Naeem, Farhad Farewar, Sayed Ataullah Saeedzai, Stephanie Simmonds

**Affiliations:** a Health in Humanitarian Crises Centre, London School of Hygiene & Tropical Medicine, Keppel Street, London, WC1E 7HT, England.; b Ministry of Public Health, Kabul, Afghanistan.

In the last two decades, the Government of Afghanistan has achieved improvements in population health despite serious episodes of violence. Between 2000 and 2015, the maternal mortality ratio reduced from 1100 to 396 deaths per 100 000 live births^1^ and under-five mortality from 257 to 55 per 1000 live births.^2^

The high level of insecurity in some provinces has had a negative effect on the provision and coverage of health services, especially for child vaccination. However, thanks to the concerted efforts of all health system actors, between 2000 and 2015 all provinces in the country increased the coverage of maternal and child health services.^3^ Despite these improvements, significant differences exist in terms of health outcomes and coverage of health services, for instance between the poorest and the wealthiest populations, between rural and urban areas, and between provinces.^4^

Afghanistan faces several health challenges, including a high burden of communicable diseases, increase of noncommunicable diseases, maternal morbidity and mortality, injuries and, in many provinces, groups of populations with low nutritional status among children.^5^ Among noncommunicable diseases, ischaemic heart disease, congenital defects and cerebrovascular disease rank among the leading causes of premature mortality.^6^ In 2016, injuries from armed conflict and road injuries ranked respectively second and fifth as causes of premature mortality. Deaths from conflict rose by almost 1100% between 2005 (1711 deaths) and 2017 (19 735 deaths).^6^ A total of 52.0% civilian fatalities (880 deaths out of 1692) were caused by suicide and complex attacks during the first half of 2018.^7^ Furthermore, according to the 2015 Afghanistan National Drug Use Survey, almost 3 million Afghans abuse from substances, that is, about 11.0% of the total population.^8^

## Health-care response

In 2001, the public health ministry started to rebuild the health system and determining how best to address the key health challenges in the country. Maternal mortality and child mortality rates were the highest in the world.^9^ In 2002 and 2003, the ministry designed a unique package of health services that helped bring cohesion among health stakeholders in what was then a fragmented health system. Towards the end of 2003, the ministry, with the support of international partners, put in place the Basic Package of Health Services for primary health care throughout the country. This initiative was followed in 2005 by the Essential Package of Health Services for hospitals up to provincial level.

In 2018, the ministry and health economists from the International Expert Committee advising the ministry estimated that in 2016, 235 million United States dollars (US$) were spent on the two packages. The basic package accounted for 72.0% (US$ 172 million) of total spending, whereas the essential package accounted for around 28.0% (US$ 63 million) of total spending (Abou Jaoude GJ et al., University College London, unpublished data, 2018 Oct 13). That same year, maternal and child health accounted for around 45% (US$ 77 million) of the basic package spending. Both packages averted an estimated 1 million disability adjusted life years (DALYs), and around 5.5% of the estimated burden of disease in Afghanistan (18 million DALYs). Almost 60.0% (605 000) DALYs averted by the two health packages are for maternal and child health.

The current health system in Afghanistan is mainly funded by households through direct out-of-pocket expense, which covers for 77.4% of the total health expenditure.^10^ According to the finance ministry, more than half (52.0%) of the national budget is funded by foreign aid, 44.8% by domestic revenue and 3.2% by national loans. From the total government budget, 4.0% is allocated to the public health ministry, of which about 80.0% is funded by donors. The ministry’s budgetary prospect exercise showed that there is limited room for expansion of the health service package, even in the best-case scenario.^11^

### Revision of the packages

In 2017, the ministry decided that both packages had to be revised, considering the increased incidence of noncommunicable diseases and injuries due to road incidents and conflict, the international drive towards universal health coverage (UHC) and the publication of the third edition of the Disease Control Priorities.

Between 2017 and 2018, the ministry, together with national working groups from different levels of the health system aimed at reviewing the content of the existing packages of health services. They translated international evidence on cost–effectiveness of interventions and on health and disease estimates from the third edition of the disease control priorities series to the Afghan context. These series have been conceived as a global guidance for health ministries to make evidence-based decisions on priority interventions. The series also identify a highest priority package for low-income countries, as well as a more extended set of interventions for achieving essential UHC. For both the essential UHC and highest priority package, the series has estimated the effect of these intervention packages on mortality reduction, as well as their incremental and total costs.

The ministry, the national working groups and committee used the structure of the series’ volumes and chapters to create consistency between the revised package of health services and the disease control priorities interventions. In addition, the ministry discussed the series’ cross-sectoral policy interventions with other relevant ministries to adopt a harmonized multisectoral approach to achieve UHC.

Two key questions identified by the committee guided the priority setting process. First, which interventions are no longer justified as a top priority and which additional health interventions are needed? Second, how will the new package of health services be accessible to the most vulnerable and geographically isolated groups of population?

The Afghan government must tackle both the epidemiological transition and armed violence, while containing the level of out-of-pocket expenses. Priority setting in the country is about making trade-offs between different types of health interventions, including clinical services, public health interventions and interventions tackling determinants of health. Priority setting is also about choosing effective interventions while managing equity. A priority setting process usually takes place in an environment where societal values are at stake and where tensions exist between different perspectives and interests. This process requires legitimacy to be accepted. As a result, all the ministry’s decisions were documented and justified with transparent arguments and criteria.

#### A multicriteria approach

The public health ministry adopted a multicriteria approach^8^ to have a fair and transparent priority setting process. This approach is based on the following principles: (i) use of the latest global and national evidence on burden of disease and cost–effectiveness of interventions (including the third edition of the disease control priorities); (ii) agreement by all stakeholders on well-defined selection criteria; (iii) transparent and documented process of selecting interventions; and (iv) recognition by all health systems actors that decisions made are reasonable, combining both analysis of evidence and expert discussions.

The selection criteria defined at the second committee meeting in May 2018 includes the following: (i) effectiveness: what has been proven to work? (ii) local feasibility: do local resources exist to deliver? Are there staff in place? Are they trained? Is the intervention supported by existing infrastructure? (iii) affordability: are new drugs and equipment required? Is there a large setup cost? and (iv) equity: will the intervention improve access to care? For whom?

The committee and the ministry also agreed on a set of priority conditions and risks factors to address the current burden of disease in Afghanistan. The priority conditions included communicable diseases, reproductive, maternal, newborn and child health, injuries (due to conflict and road injuries), mental health (substance use, suicide, posttraumatic stress disorder), cardiovascular diseases (heart attack, stroke), undifferentiated emergency presentation (difficulty breathing, shock, meningitis, diarrheal disease, lower respiratory diseases) and diabetes. The priority risk factors identified included undernutrition, unhealthy diet, smoking, poor access to water, lack of sanitation and hygiene, air pollution and hypertension.

The revised package of health services for Afghanistan, the Integrated Package of Essential Health Services, has helped the ministry to identify health interventions already delivered by health facilities, but not made explicit in the previous packages. The new package has also allowed the ministry to: (i) define interventions throughout the whole health sector, which will help service providers to identify their role and responsibility at each level of the health system; (ii) develop a unique package of health services from the community level to provincial hospitals; and (iii) clearly design the referrals links between each level of the health system. The only new interventions explicitly added to the new package are the basic management of diabetes and hypertension, emergency care and palliative care.

The implementation of the new package of health services, officially launched in Kabul on 5 January 2019 by the Minister of Health of Afghanistan and the Director-General of the World Health Organization, will help make the health system more resilient, especially to disease and injury shocks. Implementation of the new package will hopefully result in ownership of its services, implementation of priority intersectoral interventions and improvement in financial risk protection for the poor. The priority setting process is seen as a driver for positive change in the health system.
